# A Web Application for Predicting Drug Combination Efficacy Using Monotherapy Data and IDACombo

**DOI:** 10.26502/jcsct.5079218

**Published:** 2023-12-08

**Authors:** Yunong Xia, Alexander L. Ling, Weijie Zhang, Adam Lee, Mei-Chi Su, Robert F. Gruener, Sampreeti Jena, Yingbo Huang, Siddhika Pareek, Yuting Shan, R. Stephanie Huang

**Affiliations:** 1 Department of Experimental and Clinical Pharmacology, University of Minnesota, Minneapolis, MN 55455, USA; 2 Harvey Cushing Neuro-oncology Laboratories, Department of Neurosurgery, Hale Building for Transformative Medicine, 4th and 8th floor, Brigham and Women’s Hospital; 60 Fenwood Road, Boston, MA 02116.

**Keywords:** IDACombo, Independent drug action, High-throughput drug screens, Drug combinations, Drug repurposing, Cancer, Computational biology

## Abstract

We recently reported a computational method (IDACombo) designed to predict the efficacy of cancer drug combinations using monotherapy response data and the assumptions of independent drug action. Given the strong agreement between IDACombo predictions and measured drug combination efficacy in vitro and in clinical trials, we believe IDACombo can be of immediate use to researchers who are working to develop novel drug combinations. While we previously released our method as an R package, we have now created an R Shiny application to allow researchers without programming experience to easily utilize this method. The app provides a graphical interface which enables users to easily generate efficacy predictions with IDACombo using provided data from several high-throughput cell line screens or using custom, user-provided data.

## Availability and Implementation

The R Shiny app itself can be accessed at https://oncotherapyinformatics.org/idacombo/. The source code for the R Shiny app is available on GitHub (https://github.com/yunong-xia/IDACombo-Shiny-App). The R package IDACombo upon which this app is based is also available on GitHub (https://github.com/Alexander-Ling/IDACombo/).

## 1. Introduction

Despite the vital role of combination drug therapy in cancer treatment, it is impractical to exhaustively screen the huge number of possible drug combinations experimentally. As such, there are concentrated efforts to develop computational algorithms which can accurately predict the efficacy of drug combinations. To this end, we recently developed IDACombo, a computational method which uses pre- clinical measurements of monotherapy cell response data to predict the efficacy of drug combinations under the assumptions of independent drug action (IDA) [1]. IDA hypothesizes that the baseline efficacy of a combination therapy is simply the effect of the single best drug in the combination, and observed clinical efficacies from a large number of trials spanning many different cancer drug combinations were shown to be consistent with IDA rather than drug additivity or synergy [2, 3]. Given the demonstrated clinical relevance of the predictions produced with IDACombo, we previously released the algorithm as an R package. In this work, we created a web-based app for IDACombo which allows researchers with/without computational background to generate drug combination efficacy predictions using a graphical user interface.

## 2. Underlying software and hosting

The IDACombo app was created using the shiny v1.5.0 package [4] in R v4.0.3 [5] along with the following packages: shinydashboard v0.7.1 [6], shinyhelper v0.3.2 [7], DT v0.16 [8], tidyverse v1.3.0 [9], IDACombo v1.0.2 [1], shinycssloaders v1.0.0 [10, 11], shinyWidgets v0.5.4 [12], rgl v0.100.54 [13], car v3.0–9 [14], data.table v1.13.6 [15], shinyjs v2.0.0 [11], shinybusy v0.2.2 [16], openxlsx v4.2.3 [17], ggplot2 v3.3.3 [9], gridExtra v2.3 [18], promises v1.1.1 [19], future v1.21.0 [20], doFuture v0.12.2 [20], ipc v0.1.3 [21], and memuse v4.1–0 [22, 23]. The app is hosted on virtual machines (VMs) purchased from DigitalOcean (https://www.digitalocean.com/). Each VM is running Ubuntu 20.04.1 LTS (GNU/Linux 5.4.0– 51-generic x86_64) with 8 virtual CPUs, 16 GB RAM, and 100 GB disk space. Traffic is split across VMs using a load balancer. Connections within each VM are handled via Apache server 2.4 and shiny server v1.5.15.953-amd64.

## 3. Interpreting IDACombo’s Output Metrics

This app provides predicted drug combination efficacies as both summary plots and downloadable tables. The efficacy metrics used in the plots and tabular outputs are briefly described below, with descriptions of the actual plots themselves being provided later in this manuscript alongside descriptions of the analyses to which each plot is relevant. Note that equations and complete descriptions for these metrics can be found in the original IDACombo manuscript [1].

### 3.1 Average Efficacy within a Population:

Efficacy metrics for user provided datasets can be any measure of the effect of a drug on the models being tested. For the pre-provided datasets, viability is used as the efficacy metric, with a viability of 0 indicating all cells died when treated with a compound/combination and a viability of 1 indicating all cells remained alive relative to an untreated control. It should be noted that IDACombo generates predictions of efficacy at the population level rather than the individual level, so reported efficacies are averages over all cell lines/models used in a prediction rather than efficacies for individual cell lines/models.

### 3.2 Hazard Ratios (HRs):

HRs represent the relative risk of model systems remaining alive following treatment with a drug combination as compared to a control therapy. With provided datasets (or when custom datasets have been uploaded with the “Lower Efficacy Is Better Drug Effect” option selected), HRs are calculated by simply dividing the mean test treatment efficacy across the selected population of cell lines/models by the mean control treatment efficacy. As such, HRs of 1 indicates that the test therapy provides no benefit relative to the control therapy, whereas lower HRs indicate that the test therapy is more effective than the control therapy. When comparing drug combinations which all include a common control therapy (i.e. comparing all of the possible drugs you could combine with 5-fluorouracil to 5-fluorouracil treatment alone), HRs should be the preferred metric of combination efficacy. It is important, however, to look at HRs relative to both the control therapy and the drug(s) being added to that therapy, as it is possible that adding a drug to a therapy significantly improves efficacy relative to the control therapy but is no better than the efficacy achieved using the added drug as a single agent.

Importantly, when datasets have been uploaded without the “Lower Efficacy Is Better Drug Effect” option selected (i.e. when lower efficacy metric values indicate higher model system survival), HRs are calculated by dividing (1 – mean test treatment efficacy) by (1 – mean control treatment efficacy). As such, IDACombo HRs are only well defined when used with efficacy metric that range from 0 to 1, and care should be taken to avoid situations in which mean treatment efficacies become negative.

### 3.3. IDAComboscores:

The IDAComboscore provides a metric by which combinations that do not share a common control therapy can be compared. Higher IDAComboscores indicate better combination efficacy, with the aim of maximizing additional cell death caused by a combination relative to its relevant control therapy while also minimizing the HR of a combination relative to its control therapy.

## 4. User interface:

### 4.1 Selecting a Dataset to Generate Predictions With:

Users can use the “Dataset Loader” tab to choose whether to generate drug combination predictions using one of the pre-provided drug screening datasets (i.e. CTRPv2, GDSC1, GDSC2, or PRISM-Repurposing, see Table 1) or using their own dataset. Custom datasets should be formatted according to the instructions provided in the app, and a sample file can be downloaded and edited to ensure correct formatting.

Pre-provided datasets were generated using the Simplicity web app (https://oncotherapyinformatics.org/simplicity/) [24] which provides a simple graphical interface with which users can explore and perform calculations with data from high-throughput drug screens in cancer cell lines. While cell line filtering options in the IDACombo app have been kept to a minimum to avoid unnecessary difficulty for users uploading custom datasets to the website, the Simplicity app provides extensive filtering capabilities for drugs and cell lines and can generate datasets formatted for direct use with the IDACombo app.

### 4.2 Generating Drug Combination Efficacy Predictions

The IDACombo app provides three basic ways to generate drug combination efficacy predictions: (1) 2-Drug predictions, (2) Control Plus One predictions, and (3) Test vs Control predictions. Each of these approaches are contained in a separate tab within the app, with subtabs providing focused or batch functionalities. Brief descriptions for each approach are provided below.

#### 4.2.1. 2-Drug tab:

Allows users to generate predictions for 2-drug combinations at a range of concentrations.

**Figure 1:**
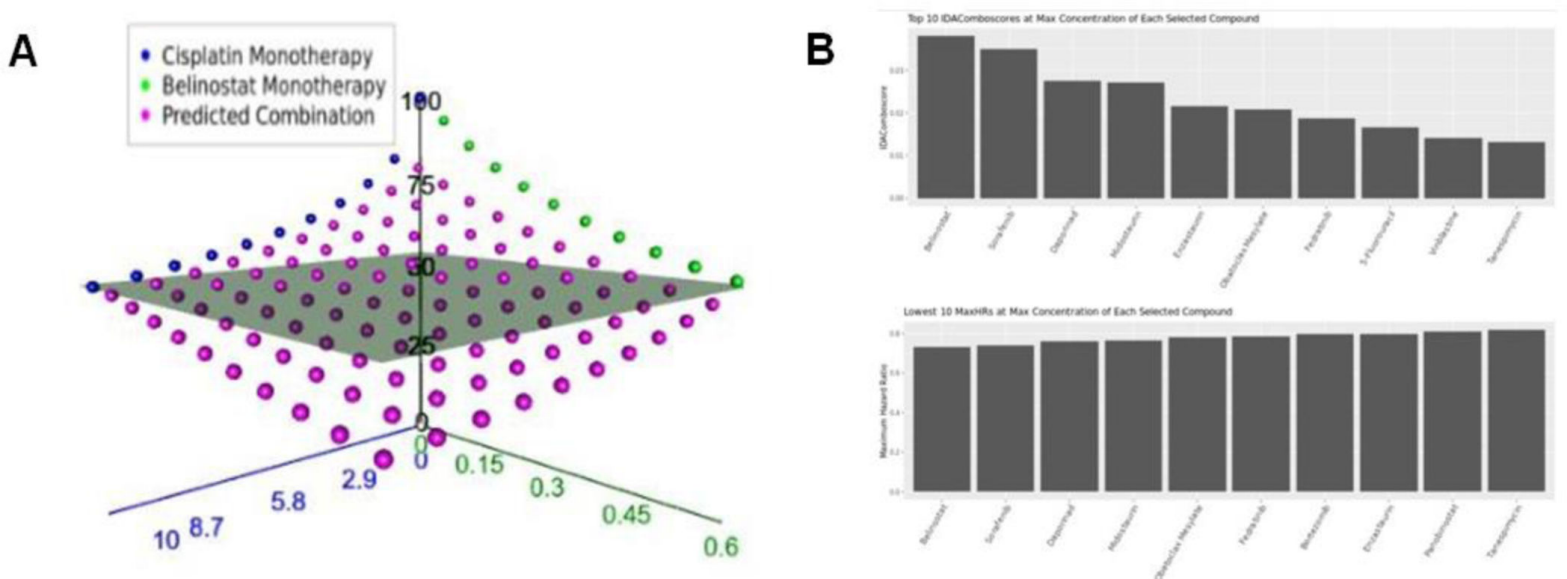
Automatic visualization of IDACombo predictions. A) 3-d plot of measured of measured monotherapy (blue and green points) and predicted combination (purple points) average viability values for the combination of cisplatin + belinostat across a range of concentrations in 800 cancer cell lines. The grey plane represents the best achievable viability by either monotherapy. Plot was produced using the 2-Drug/Focused tab of the IDACombo app. B) Bar-plots showing top candidates for 2-drug combination with cisplatin. Plots show top IDAComboscores (top panel) and maximum hazard ratios (bottom panel) when predictions were generated using all available GDSC1 cell lines for cisplatin + any all drugs with available Csustained concentrations in the GDSC1 dataset. Predictions were only generated using concentration ranges between 0 and Csustained for each compound.

- Focused: Generates predictions for the efficacy of a single 2-drug combination across multiple, user selected, concentrations of each drug. Results are provided as an interactive 3D plot of predicted average efficacy across selected cell lines at each drug-concentration combination (Figure 1A) and as a downloadable tabular output.
- Batch Processing: Generates predictions for many 2-drug combinations across all available concentrations for each drug within the selected cell line population. Results are provided as barplots of the 10 combinations predicted to be most efficacious as ranked by IDAComboscore or HR when combined at the maximum concentrations of each drug (Figure 1B). Results are also provided as a downloadable tabular output.

#### 4.2.2. Control Plus One tab:

Allows users to generate predictions of the added benefit from adding a single additional drug to a control treatment consisting of one or more drugs combined at single, user specified, and concentrations for each drug.

- Focused: Generates predictions for adding a single specified compound (at multiple, user selected, concentrations) to an existing control treatment. Results are provided as scatterplots showing how combination efficacy (i.e. viability for pre-provided datasets), HRs (vs. control treatment or vs the drug being added), and IDAComboscores are impacted by combining the drug of interest with the control treatment at different concentrations. Results are also provided as a downloadable tabular output.
- Batch Processing: Generates predictions for adding many drugs (one at a time) to an existing control treatment at all available concentration for the drugs being added. Results are provided as barplots of the 10 combinations predicted to be most efficacious as ranked by IDAComboscore or HR when combined at the maximum concentration of each added drug. Results are also provided as a downloadable tabular output.

#### 4.2.3. Test vs Control tab:

Allows user to generate predictions for whether a test therapy consisting of one or more drugs provides more efficacy than a control therapy consisting of one or more drugs. Results are provided as a downloadable tabular output.

All calculation tabs in the app allow users to specify drug concentrations and cell lines to use when generating predictions. Cell lines can be filtered by general cancer type using a drop- down menu, and check-box options can be selected to modify how calculations are performed. Each option has a clickable question mark next to it which provides details for what each option does.

## 5. Primary use cases

### 5.1 Finding the best single drug to combine with a single drug of interest (use case for batch use of 2-Drug function):

A common use case may be when a researcher has a single drug of interest and would like to identify other drugs that could be efficaciously combined with this drug. This can be achieved by using the batch functionality of the “2-Drug” function in our app. After selecting a dataset in the “Dataset Loader” tab, users should navigate to the “Batch Processing” dropdown option under the “2-Drug” tab. In this page, a user can specify a drug of interest and multiple drugs to add. The app will predict efficacies for all 2-drug combinations between the selected drug of interest and drugs to add using all available concentrations for each drug in the loaded dataset. Note that users must also select which cell lines IDACombo should use to generate the predictions. After selecting these options and pressing “RUN”, a table of the prediction results will be generated which can be navigated within the app or downloaded using the “Download DataTable” button. Batch use of the 2-Drug function can be useful not only as a way for finding an effective 2-drug combination therapy which includes a drug of interest, but also as a means of identifying which drug classes (i.e. mechanisms of action) target different cell populations than a user’s drug of interest. This is because, since IDACombo is built upon IDA, the efficacious combinations identified by IDACombo must consist of combinations of drugs which effectively target at least partially non-overlapping cell populations.

### 5.2 Finding the best drug to add to a standard combination therapy to improve its efficacy (use case for batch processing in Control Plus One function):

Another common use case may be when a researcher would like to identify drugs which can be added to an existing combination therapy to improve its efficacy. This can be achieved using the “Batch Processing” interface under the “Control Plus One” function tab. To perform the analysis, users define the control therapy by selecting the drugs and drug concentrations of the therapy. Users then select which drugs to add to the therapy and which cell lines to use when predicting combination efficacy. Predictions can be generated by pressing “RUN”, and the app will generate efficacy predictions for each combination of the control therapy + additional drug using all available concentrations for each drug that is being added to the control therapy. As with the 2-Drug function, these predictions will be output as a table which can be navigated within the app or downloaded as a tab-delimited text file. Several plots will also be generated to visualize the drugs that are predicted to combine best with the control therapy.

### 5.3 Estimating HR for control therapy vs test therapy as might be tested in a clinical trial (use case for non-batch use of Test vs Control function):

The last use case we will highlight is when a researcher may wish to compare a test therapy to a control therapy which may or may not share overlapping drugs with the test therapy. In the page of Test Vs Control, a user can specify a test treatment and a control treatment, as well as cell lines which can also be selected based on cell line subgroups as we previously discussed. The HR computed by IDACombo can indicate whether the test therapy is predicted to have improved efficacy compared to the control therapy. Note that this is not yet validated to be useful when the test therapy does not contain all of the drugs in the control therapy (i.e. when comparing two unrelated therapies such that the test therapy is not the control therapy plus one or more additional drugs).

## 6. Video tutorials

The following video tutorials have been created to facilitate use of the IDACombo app:

1. How the IDACombo algorithm works
2. How to use this app
3. How to generate custom datasets for this app using the Simplicity app

Links to these videos can be found on the introduction page of the IDACombo web app (https://oncotherapyinformatics.org/idacombo/).

## Conclusion

The IDACombo web app allows non-programmatic users to predict drug combination efficacy using monotherapy drug-screening data from cancer cell lines. The predictions of this algorithm have been validated against both pre-clinical and clinical studies of drug combination efficacy (Ling and Huang, 2020). The drug screening data necessary to use IDACombo has been built into the app, and users can upload custom datasets to easily convert their own monotherapy drug screening data into clinically meaningful predictions of drug combination efficacy in cancer.

## Figures and Tables

**Table 1:** Pre-provided drug screening datasets datasets available for use with the IDACombo app

Dataset	Version	Screening Location	Assay Method	Plate Format	Treatment Duration	# of Compounds	# of Cell Lines	References
CTRPv2	v2	Broad Institute	CellTiter-Glo	1536 well	72 hours	544	887	(Basu *et al.*, 2013; Seashore-Ludlow *et al.*, 2015; Rees *et al.*, 2016)
GDSC1	v8.2	MGH or Sanger	Resazurin or Syto60	96 or 384 well	72 hours	343	987	(Iorio *et al.*, 2016; Yang *et al.*, 2013; Garnett *et al.*, 2012)
GDSC2	v8.2	Sanger	CellTiter-Glo	1536 well	72 hours	192	809	
PRISM-Repurposing	Accessed 4/6/2020	Broad Institute	HTS-PRISM or MTS-PRISM	384 well	120 hours	1446	481	(Corsello *et al.*, 2020)
